# Prognostic significance of neutrophil-to-lymphocyte ratio in diffuse large B-cell lymphoma: A meta-analysis

**DOI:** 10.1371/journal.pone.0176008

**Published:** 2017-04-25

**Authors:** Jin Wang, Xu Zhou, Yu Liu, Zheng Li, Xiang Li

**Affiliations:** Hematology Department of Research Institute of Surgery, Daping Hospital, Third Military Medical University, Chongqing, China; Hokkaido Daigaku, JAPAN

## Abstract

**Background:**

Neutrophil-to-lymphocyte ratio (NLR) has been investigated as a prognostic marker in patients with diffuse large B-cell lymphoma (DLBCL); however, the results remain controversial. This study aimed to explore the association between NLR and survival outcomes and clinicopathological factors in DLBCL.

**Methods:**

Relevant studies were retrieved by searching PubMed, Embase, Web of Science, and China National Knowledge Infrastructure (CNKI) databases. The last search was updated on February 17, 2017. Hazard ratios (HRs) and odds ratios (ORs) and their 95% confidence intervals (CIs) were used as effective measures in the meta-analysis. Random-effects models and fixed-effects models were used for analyses. Meta-regression was performed. Publication bias was assessed using Begg’s test. Stata version 12.0 was used for all analyses.

**Results:**

A total of 9 studies with 2297 patients were included in the meta-analysis. The pooled results showed that NLR was a significant indicator for poor overall survival (OS) (HR = 1.84, 95% CI = 1.52–2.22, p<0.001) and poor progression-free survival (PFS) (HR = 1.64, 95% CI = 1.36–1.98, p<0.001). NLR remained a significant biomarker for OS and PFS regardless of location, sample size or cut-off value. In addition, high NLR was also associated with Ann Arbor stage (OR = 2.09, 95% CI = 1.14–3.81, p = 0.017), lactate dehydrogenase level (OR = 2.74, 95% CI = 1.16–6.46, p = 0.021), extranodal disease (OR = 1.63, 95% CI = 1.06–2.52, p = 0.027), and International Prognostic Index score (OR = 2.44, 95% CI = 1.03–5.08, p = 0.043). However, NLR was found to have no significant association with sex (OR = 0.89, 95% CI = 0.71–1.11, p = 0.29), age (OR = 1.18, 95% CI = 0.94–1.48, p = 0.152), European Cooperative Oncology Group performance status score (OR = 1.78, 95% CI = 0.71–4.46, p = 0.217), or presence of B symptoms (OR = 1.56, 95% CI = 0.7–3.48, p = 0.278).

**Conclusion:**

In conclusion, our meta-analysis demonstrated that NLR has a strong association with worse OS and PFS in patients with DLBCL. NLR could be recommended as an inexpensive prognostic biomarker in DLBCL.

## Introduction

Diffuse large B-cell lymphoma (DLBCL) is the most common lymphoid malignancy in adults, accounting for approximately 20% of newly diagnosed lymphoid neoplasms [1]. DLBCL accounts for 31% of all non-Hodgkin’s lymphomas (NHL) in Western countries[2]. DLBCL is biologically and clinically heterogeneous and is typically treated with an R-CHOP (rituximab, cyclophosphamide, doxorubicin, vincristine, and prednisone) strategy [3]. Approximately 60–70% of DLBCL patients are curable using various regimens whereas other patients fail to respond to chemotherapy or have poor long-term survival outcomes [4]. Some indexes such as the International Prognostic Index (IPI) and gene expression profiling (GEP) can be used to identify risk patients [5, 6]. However, these parameters either lack the accuracy for prognosis or are hard to obtain in everyday clinical practice. Therefore, simple, inexpensive, and easily available prognostic biomarkers are urgently needed.

Inflammation often already exists in the tumor microenvironment before tumor occurrence and continues to facilitate tumor progression [7, 8]. Systemic inflammatory responses caused by and accompanied by tumorigenesis could provide information for prognostication. A series of prognostic indexes based on laboratory test results have emerged as objective and inexpensive indicators [9]. Such parameters include C-reactive protein (CRP), neutrophil-to-lymphocyte ratio (NLR), platelet-to-lymphocyte ratio (PLR), lymphocyte-to-monocyte ratio (LMR), and modified Glasgow Prognostic Score (mGPS)[10–15], of which, NLR was found to be related with worse survival outcomes in a variety of tumors [16], including DLBCL [12, 13, 17, 18]. However, previous studies have reported controversial results concerning the prognostic value of NLR in DLBCL [12, 17–20]. The conflicting results may be due to small sample sizes and heterogeneous patients in individual studies. To comprehensively evaluate NLR in DLBCL, a meta-analysis was performed by aggregating data from relevant studies. In this meta-analysis, we investigated the relationship between NLR and overall survival (OS) and progression-free survival (PFS); in addition, we also explored the association between NLR and different clinicopathological factors in DLBCL.

## Materials and methods

This meta-analysis was carried out in accordance with Preferred Reporting Items for Systematic Reviews and Meta-Analyses (PRISMA) statement [21].

### Literature search

A systematic literature search was performed by using the databases of PubMed, Embase, Web of Science, and China National Knowledge Infrastructure (CNKI) for all relevant studies. There was no language restriction. The last literature search was updated on February 17, 2017. The following terms were used in the search: “NLR,” “neutrophil-lymphocyte ratio,” “neutrophil to lymphocyte ratio,” “diffuse large B-cell lymphoma,” “lymphoma, large B-cell, diffuse” [MeSH Terms], and DLBCL. The references in the relevant studies were also screened for possible inclusions.

### Selection criteria

The inclusion criteria were as follows: (a) NLR was obtained from a hematological test before treatment; (b) the diagnosis of DLBCL was pathologically confirmed; (c) the relationships between NLR and survival including OS or PFS were investigated or sufficient data were provided; (d) studies were published as full-text articles in English or Chinese. Literature falling under the following category was excluded: (a) reviews, meeting abstracts, letters, and duplicate studies; (b) irrelevant studies; (c) animal studies; and (d) studies without sufficient data.

### Data extraction and qualitative assessment

Data were extracted by two independent investigators from eligible studies; discrepancies were resolved by joint discussion. The following information was extracted: first author, year of publication, study location, number of patients, tumor stage, treatment regimens, research period, cut-off value, survival outcomes, and hazard ratios (HRs) and 95% confidence intervals (CIs) for OS and/or PFS. The quality of each study was evaluated by using the 9-star Newcastle-Ottawa Scale (NOS) for cohort studies[22]. Studies with scores ≥7 were considered high-quality studies.

### Statistical analysis

HRs with corresponding 95% CIs were used to evaluate the relationships between NLR and OS and/or PFS in DLBCL. Odds ratios (ORs) with 95% CIs were used to assess the strength of association between NLR and clinicopathological parameters. The heterogeneity among studies was calculated using Cochran’s Q test and Higgins I-squared statistic. P for heterogeneity <0.1 or *I*^2^ >50% was considered a significant level of heterogeneity. Both fixed effects (Mantel—Haenszel method) and random effects (DerSimonian and Laird method) models were performed to generate the pooled HRs. The random-effects model is more conservative and provides better estimates with wider confidence intervals[23, 24]. Meta-regression was also performed. Sensitivity analysis was conducted by sequential omission of each included studies. Publication bias was tested using Begg’s funnel plots. P<0.05 was considered statistically significant. All statistical analyses were performed with Stata version 12.0 (Stata Corp, College Station, TX).

## Results

### Literature selection and study characteristics

**Fig 1:**
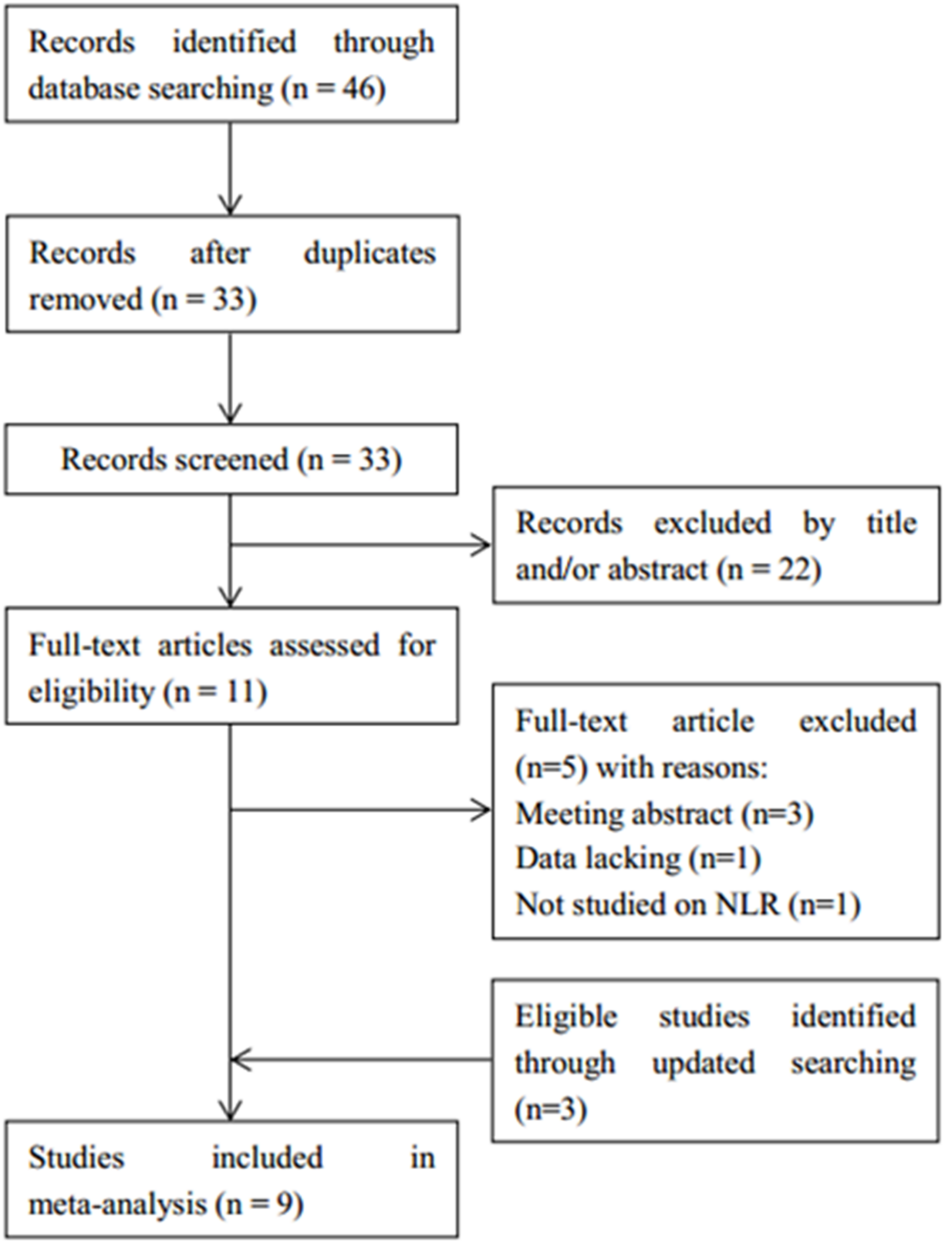
Flowchart of article selection.

The flowchart of the literature selection process is shown in Fig 1. A total of 46 studies were identified through database searching, 33 records were screened after duplicates were excluded. Then, 22 records were excluded after title and/or abstract reading. Subsequently, 11 full-text articles were evaluated for eligibility. Five studies were further excluded because they were meeting abstracts and studies with insufficient or those that did not focus on NLR. Three studies[25–27] were found to be eligible through updated searching. In total, 9 studies [12, 13, 17, 18, 25–29] with 2297 patients were included for the meta-analysis. The main characteristics of included studies are demonstrated in Table 1. The studies were published between 2010 and 2017. Sample sizes ranged from 51 to 515. Seven studies[12, 13, 17, 18, 25–27] were published in English and 2 studies [28, 29] were published in Chinese. Eight studies [12, 13, 17, 18, 26–29] investigated the association between NLR and OS and 7 studies [12, 17, 18, 25–27, 29] reported a connection between NLR and PFS. All studies obtained a NOS score of ≥7. The detailed information of quality assessment is shown in Table 2.

**Table 1:** Characteristics of included studies.

Study	Year	Region	NOS score	Sample size	Age (years) median(range)	Stage	Treatment regimen	Study period	Cut-off	Outcomes analyzed
Porrata	2010	USA	8	255	64(20–92)	I-IV	R-CHOP	2000–2007	3.5	OS, PFS
Ho	2015	Taiwan	9	148	61(16–88)	I-IV	R-CHOP	2001–2010	4.35	OS, PFS
Keam	2015	Korea	8	447	61(16–87)	I-IV	R-CHOP	2003–2010	3	OS, PFS
Melchardt	2015	Austria	8	515	65(20–92)	I-IV	R-CHOP	2004–2014	5.54	OS
Ming	2015	China	7	51	55(20–85)	I-IV	R-CHOP	2009–2013	2.32	OS
Hong	2016	Korea	8	313	56(16–86)	I-IV	R-CHOP	2008–2011	2.42	PFS
Ni	2016	China	7	57	54(14–75)	I-IV	R-CHOP	2009–2015	2.915	OS, PFS
Wang	2016	China	8	156	NR	I-IV	R-CHOP	2006–2015	3	OS, PFS
Wang	2017	China	9	355	54(18–86)	I-IV	R-CHOP	2005–2011	2.81	OS, PFS

OS = overall survival; PFS = progression-free survival; R-CHOP = rituximab, cyclophosphamide, doxorubicin, vincristine, and prednisone; NOS = Newcastle-Ottawa Scale, NR = not reported.

**Table 2:** Newcastle-Ottawa Scale for quality assessment of studies included in the meta-analysis.

Study	Selection				Comparability	Outcome			Overall
	Representativeness of the exposed cohort	Selection of the nonexposed cohort	Assessment of exposure	Outcome not present at start		Assessment of outcome	Follow-up long enough for outcomes	Adequacy of follow-up	
Porrata (2010)	☆	☆	☆	☆	☆☆	☆		☆	8
Ho (2015)	☆	☆	☆	☆	☆☆	☆	☆	☆	9
Keam (2015)	☆	☆	☆	☆	☆☆	☆	☆		8
Melchardt (2015)	☆	☆	☆	☆	☆☆	☆	☆		8
Ming (2015)	☆	☆	☆	☆	☆☆	☆			7
Hong (2016)	☆	☆	☆	☆	☆☆	☆	☆		8
Ni (2016)	☆	☆	☆	☆	☆☆	☆			7
Wang (2016)	☆	☆	☆	☆	☆☆	☆	☆		8
Wang (2017)	☆	☆	☆	☆	☆☆	☆	☆	☆	9

### NLR and OS, PFS

**Fig 2:**
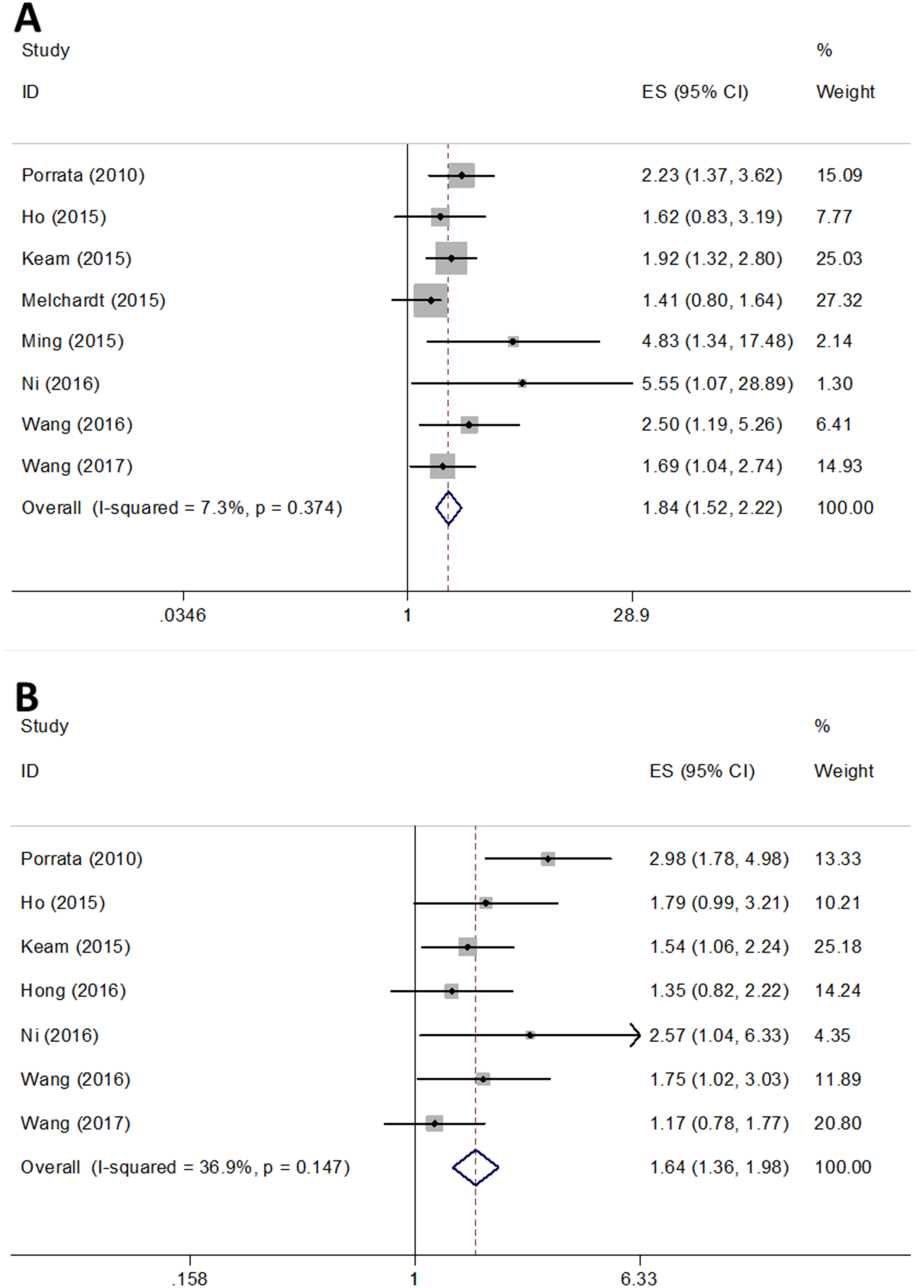
Forest plots for the estimate of NLR associated with (A) OS and (B) PFS in the meta-analysis.

Eight studies [12, 13, 17, 18, 26–29] with 1984 patients showed the relationship between NLR and OS in DLBCL. The heterogeneity tests suggested non-significant heterogeneity (*I*^2^ = 7.3%, P_H_ = 0.374; Table 3). The pooled data showed that a high NLR significantly correlated with a worse OS (HR = 1.84, 95% CI = 1.52–2.22, p<0.001, Table 3, Fig 2). For further investigation, we conducted subgroup analysis. As shown in Table 3, the results demonstrated that NLR remained a significant prognostic marker regardless of ethnicity (Asian or Non-Asian), sample size (<200 or ≥200), and cut-off value (NLR ≤3 or NLR >3). In terms of NLR and PFS, the combined results from 7 studies[12, 17, 18, 25–27, 29] with 1731 patients showed that NLR was also a factor predicting worse PFS in DLBCL (HR = 1.64, 95% CI = 1.36–1.98, p<0.001; *I*^2^ = 36.9%, P_H_ = 0.147, Table 3, Fig 2). Subgroup analysis demonstrated that NLR was still a prognostic biomarker regardless of study location, sample size, and cut-off. The results of meta-regression are shown in Table 3.

**Table 3:** Main results of meta-analysis.

Outcome	Variables	No. of studies	Heterogeneity		Fixed-effects model		Random-effects model		Meta-regression
			*I*^2^(%)	P_H_	HR (95%CI)	p	HR (95%CI)	p	p
OS	All	8	7.3	0.374	1.84(1.52–2.22)	<0.001	1.85(1.52–2.26)	<0.001	
	Ethnicity								0.424
	Asian	6	0	0.479	1.98(1.55–2.54)	<0.001	1.98(1.55–2.54)	<0.001	
	Non-Asian	2	54.9	0.136	1.66(1.24–2.22)	0.001	1.72(1.1–2.69)	0.017	
	Sample size								0.264
	<200	4	12.5	0.33	2.37(1.52–3.72)	<0.001	2.45(1.49–4.02)	<0.001	
	≥200	4	0	0.458	1.74(1.41–2.14)	<0.001	1.74(1.41–2.14)	<0.001	
	Cut-off								0.326
	≤3	5	2.9	0.39	2.04(1.57–2.67)	<0.001	2.05(1.56–2.7)	<0.001	
	>3	3	10	0.329	1.65(1.27–2.16)	<0.001	1.67(1.25–2.21)	<0.001	
PFS	All	7	36.9	0.147	1.64(1.36–1.98)	<0.001	1.69(1.32–2.15)	<0.001	
	Ethnicity								0.059
	Asian	6	0	0.612	1.5(1.23–1.84)	<0.001	1.5(1.23–1.84)	<0.001	
	Non-Asian	1	-	-	2.98(1.78–4.98)	<0.001	2.98(1.78–4.98)	<0.001	
	Sample size								0.564
	<200	3	0	0.756	1.88(1.3–2.71)	0.001	1.88(1.3–2.71)	0.001	
	≥200	4	63.6	0.041	1.57(1.26–1.95)	<0.001	1.61(1.11–2.33)	0.011	
	Cut-off								0.083
	≤3	5	0	0.526	1.47(1.18–1.82)	<0.001	1.47(1.18–1.82)	<0.001	
	>3	2	39.5	0.199	2.39(1.62–3.51)	<0.001	2.35(1.43–3.88)	0.001	

### NLR and clinicalpathological features

**Fig 3:**
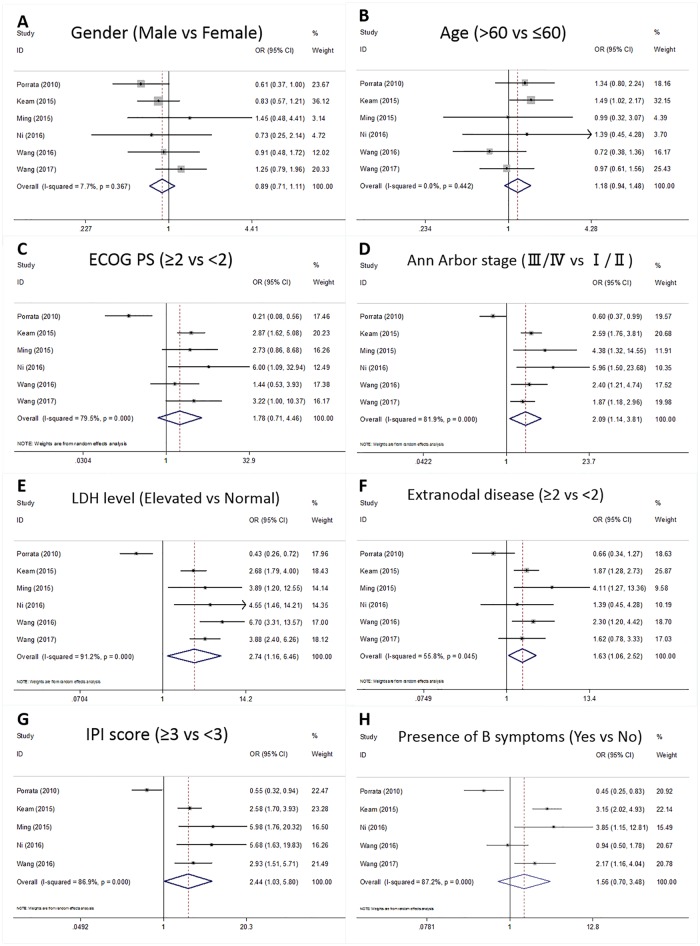
Forest plots for the association of NLR and (A) sex, (B) age, (C) ECOG PS, (D) Ann Arbor stage, (E) LDH level, (F) extranodal disease, (G) IPI score, and (H) presence of B symptoms in meta-analysis.

We also comprehensively investigated the association between NLR and clinicopathological features. A total of 8 clinicopathological features were investigated, as follows: sex (male vs. female), age (>60 vs. ≤60 years), European Cooperative Oncology Group performance status score (ECOG PS; ≥2 vs. <2), Ann Arbor stage (III/IV vs. I/II), lactate dehydrogenase (LDH) level (elevated vs. normal), extranodal disease (≥2 vs. <2), IPI score (≥3 vs. <3), and presence of B symptoms (yes vs. no). The results are summarized in Fig 3. As shown in Fig 3, NLR was associated with Ann Arbor stage (OR = 2.09, 95% CI = 1.14–3.81, p = 0.017), LDH level (OR = 2.74, 95% CI = 1.16–6.46, p = 0.021), extranodal disease (OR = 1.63, 95% CI = 1.06–2.52, p = 0.027), and IPI score (OR = 2.44, 95% CI = 1.03–5.08, p = 0.043). However, NLR was found to have no significant association with sex (OR = 0.89, 95% CI = 0.71–1.11, p = 0.29), age (OR = 1.18, 95% CI = 0.94–1.48, p = 0.152), ECOG PS (OR = 1.78, 95% CI = 0.71–4.46, p = 0.217), or presence of B symptoms (OR = 1.56, 95% CI = 0.7–3.48, p = 0.278).

### Sensitivity analysis

**Fig 4:**
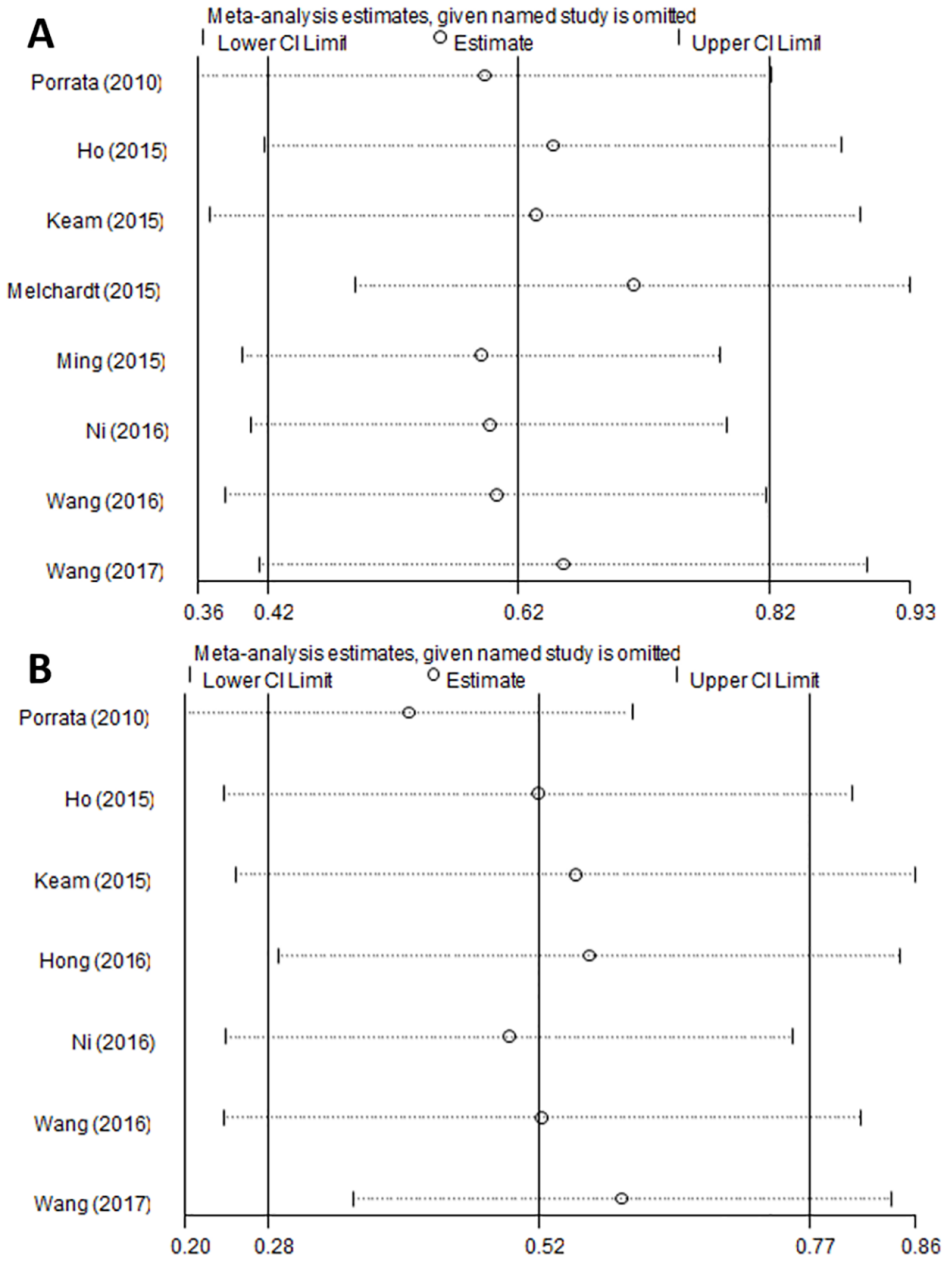
Sensitivity analysis for (A) OS and (B) PFS.

Sensitivity analysis was conducted by omitting one study at a time and analyzing the remaining studies. The results are shown in Fig 4, the results were not substantially changed, showing the reliability and stability of our results.

### Publication bias

**Fig 5:**
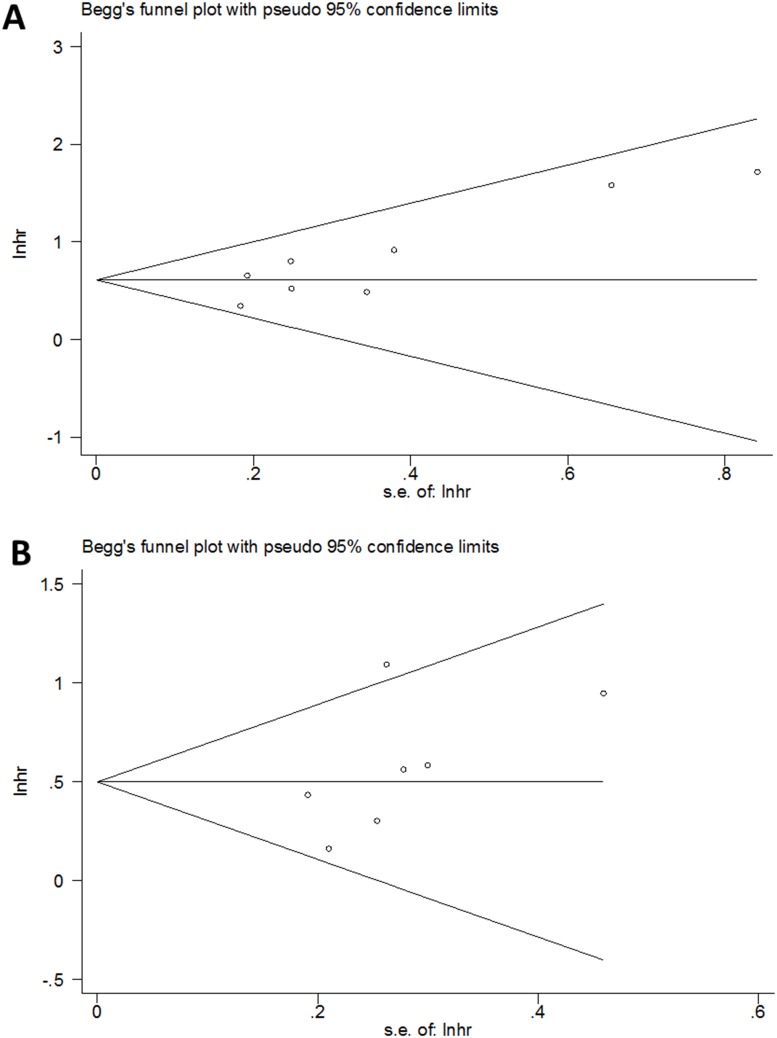
Begg’s test for (A) OS and (B) PFS.

In this meta-analysis, we introduced Begg’s funnel plot to test publication bias. As shown in Fig 5, the results suggested no significant publication bias for OS (p = 0.063) and PFS (p = 0.133).

## Discussion

Prior studies have suggested that NLR is associated with worse survival outcomes in various cancers [30–35]. As for DLBCL, the prognostic role of NLR remains controversial, which may be due to the clinically heterogeneous features of this disease. In the current meta-analysis, we aggregated data from 9 studies including 2297 patients. The results showed that NLR was correlated with poor OS (HR = 1.84, 95% CI = 1.52–2.22, p<0.001) as well as worse PFS (HR = 1.64, 95% CI = 1.36–1.98, p<0.001). In addition, in the subgroup analysis stratified by location, sample size, and cut-off value, the prognostic value of NLR remained significant. Furthermore, NLR was also associated with Ann Arbor stage, LDH level, extranodal disease, and IPI score. The findings of this study suggest that NLR is a significant prognostic marker in DLBCL; additionally, because measurement of NLR is easy and inexpensive, NLR has potential to be validated in clinical practice for DLBCL patients.

A number of studies have shown that persistent chronic inflammation could trigger tumorigenesis [7, 36, 37]. Inflammatory responses could promote angiogenesis and protect cancer cells from immune attacks [36]. In the tumor microenvironment, neutrophils secrete a variety of cytokines including interleukin-2, interleukin-10, and tumor necrosis factor α, which further promote cancer development[38]. In contrast, lymphocytes are well known to exert dominant roles in immune defense against cancer cells [39]. Lymphocytes can induce cytotoxic cell death [40]. NLR has a biological rationale because it reflects the strength of immune responses in cancer patients. A variety of meta-analyses have shown the significant prognostic value of NLR in solid tumors including lung cancer[41], gastric cancer [42, 43], hepatocellular carcinoma [44], breast cancer [32], and renal cell carcinoma [30]. The results demonstrated that NLR was associated with poor survival in various tumors, which was in accordance with results in this meta-analysis. We also noted that a meta-analysis investigating the correlation between NLR and various solid tumors[16]; however, in this meta-analysis, only 1 study regarding Hodgkin’s lymphoma [45] was included for analysis, and DLBCL was not investigated. To the best of our knowledge, this is the first meta-analysis exploring the prognostic value of NLR in patients with DLBCL. We also noted that previous meta-analyses also showed that absolute lymphocyte count and lymphocyte/monocyte ratio were prognostic markers for DLBCL[46, 47]. Feng et al. revealed that low absolute lymphocytic count has an adverse effect on outcome in DLBCL [46]. This finding is in accordance with our results because low absolute lymphocytic count leads to high NLR when the neutrophil count is fixed.

Several limitations need to be pointed out in this study. First, the sample size was relatively small. Only 9 studies were included for analysis, especially for the association between NLR and clinical parameters. The small sample size may introduce bias. Second, cut-off values of NLR were inconsistent in primary studies, which suggests a need for a uniform cut-off value in further studies. Third, publication bias examination was suboptimal in detecting publications when the included studies were fewer than 10[48]. Only 9 studies were included in this meta-analysis, although Begg’s test suggested no significant publication bias. This could not rule out the possibility of publication bias because insufficient studies were included. Fourth, meta-regression was performed although meta-regression is most suitable for analyses that include >10 cohorts[49]. Eight studies were included for OS and 7 studies were for PFS; therefore, the results of meta-regression need to be treated with caution. Fifth, the confidence intervals for *I*^2^ were not reported, which could be useful for heterogeneity estimates[50].

In conclusion, this meta-analysis indicated that elevated NLR correlated with poor OS and poor PFS in patients with DLBCL. In addition, NLR was also strongly associated with Ann Arbor stage, LDH level, extranodal disease, and IPI score. The results suggested that NLR could be recommended as an inexpensive prognostic biomarker in clinical practice for DLBCL. However, due to the limitations mentioned above, further large-scale studies are needed to confirm our results.

## Supporting information

S1 Table PRISMA checklist. (DOC)
